# Hospital length of stay among COVID-19-positive patients

**Published:** 2021-06-05

**Authors:** Tze Chiam, Keshab Subedi, David Chen, Eric Best, Federica B. Bianco, Gregory Dobler, Mia Papas

**Affiliations:** ^1^Value Institute, ChristianaCare, Newark, Delaware; ^2^School of Public Affairs, Penn State University - Harrisburg, Middletown, Pennsylvania; ^3^Department of Physics and Astronomy, University of Delaware, Newark, Delaware; ^4^Biden School of Public Policy and Administration, University of Delaware, Newark, Delaware

**Keywords:** COVID-19, length of stay, comorbidities

## Abstract

**Background and Aim:**

This study aims to determine COVID-19 patient demographics and comorbidities associated with their hospital length of stay (LOS).

**Methods:**

Design: Single-site, retrospective study. Setting: A suburban 700-bed community hospital in Newark, Delaware, USA. Patients: Patients admitted to the hospital from March 11, 2020, to August 11, 2020, with a positive COVID-19 status. We followed a time-to-event analysis approach and used Kaplan–Meir curves and log-rank tests for bivariate analyses, and an accelerated failure time model for a multivariable model of hospital LOS.

**Results:**

Six hundred and eighty-seven patients discharged alive (mean [SD] age, 60.94 [18.10] years; 339 men [49.34%]; 307 Black/African-American [44.69%]; and 267 White [38.86%]) were included in the investigation. Bivariate analysis using Kaplan–Meir curves showed that patients’ age, sex, ethnicity, insurance type, comorbidity of fluid and electrolyte disorder, hypertension, renal failure, diabetes, coagulopathy, congestive heart failure, peripheral vascular disease, neurological disorder, coronary artery disease, and cardiac arrhythmias to be significantly associated with LOS (*P*<0.05). In the multivariable analysis, patients’ age, sex, ethnicity, number of Elixhauser comorbidities, and number of weeks since onset of the pandemic was significantly associated with LOS (*P*<0.05). Fluid and electrolyte disorder is the only comorbidity independently associated with LOS after adjusting for patients’ age, sex, race, ethnicity, number of Elixhauser comorbidities, and weeks since onset of pandemic.

**Conclusion:**

COVID-19 patients LOS vary based on multiple factors. Understanding these factors are crucial to improving the prediction accuracy of COVID-19 patient census in hospitals for resource planning and care delivery.

**Relevance for patients:**

Understanding of the factors associated with LOS of the COVID-19 patients may help the care providers and the patients to better anticipate the LOS, optimize the resources and processes, and prevent protracted stays.

## 1. Introduction

Reports of an outbreak of a new pneumonia-like virus originated from Wuhan, China, in the Fall of 2019 [1]. The identified infectious agent, a novel coronavirus known as SARS-CoV-2, spreads rapidly and by mid-January cases was identified beyond China in Japan, Thailand, and South Korea [2]. SARS-CoV-2 causes a respiratory illness we now commonly refer to as COVID-19 [3]. The first case of COVID-19 in the United States occurred in January 2020 and, by the end of the month, the World Health Organization declared the outbreak a global public health emergency with 9000 cases worldwide [4,5]. Over the past few months, we witnessed the destabilization of the world economic markets and watched in disbelief as overrun hospitals and soaring death rates in Italy, Spain, Brazil, and the United States became warning flares for the rest of the world. As of October 2020, the world continues to be engulfed in a pandemic totaling over 40 million confirmed cases and 1.1 million deaths with 8.2 million cases and 220,000 deaths in the United States [6].

Due to the lack of knowledge in managing the spread of COVID-19, health-care systems were overwhelmed by the drastic increases of patients seeking medical care. With the recent discovery of new variants [7], health-care systems have raised concerns on further increases of patients needing care.

To provide insights into prevent such a phenomenon, various models are developed to predict ICU admission, hospital admission, and ventilators needed [8-11]. Although they differ in interfaces, they are fundamentally based on the epidemiologic susceptible, infectious, and recovered model commonly used to understand the spread of infectious diseases [12]. Within these models, patient hospital length of stay (LOS) is a crucial variable. Defined as the time elapsed between a patient’s admission to discharge from the hospital, the LOS of COVID-19 patients is broadly categorized in the literature into three groups: Patients who need ventilators in the ICU, patients in the ICU who do not need ventilators, and patients in the medical unit.

To date, most LOS estimates come from investigations conducted in China [13]. A systematic review found that of the 52 studies examining LOS, 46 (88%) were based in China. The median hospital LOS ranged from 4 to 53 days and 6 to 12 days for ICU stays. Studies from outside China found that median hospital LOS ranged from 4 to 21 days and 4 to 19 days for ICU stays. The unique nature of hospital systems makes it critically important to understand LOS for a wide variety of populations in multiple countries. In addition, little is known about the factors that influence LOS for COVID-19 patients. In non-COVID-19 populations, hospital LOS is impacted by comorbidities [14] and demographics [15]. There are significant differences between China and the US in terms of demographics and comorbidities, illustrating the need for information on LOS for COVID-19 patients in the US [16,17]. As new pharmacological interventions and improved medical management for COVID-19 patients become available, it is also critical to understand how LOS estimates change overtime. Due to the new variants of the virus that causes COVID-19, the ability to predict LOS will be crucial as higher LOS is also associated with cost [18] and capacity utilization [19].

Therefore, the goals of this study are: (1) to describe time trends in hospital LOS among COVID-19 patients, (2) to investigate covariates associated with LOS for more accurate prediction of COVID-19 hospital census, and (3) to provide insight into the impact of COVID-19 patients’ comorbidities on LOS for improved clinical management.

## 2. Methods

### 2.1. Study design and setting

A retrospective analysis of electronic health records (EHRs) of patients diagnosed with COVID-19 admitted to ChristianaCare before June 30, 2020, was conducted. ChristianaCare is one of the largest health-care providers in the mid-Atlantic with a Level 1 trauma center serving all of Delaware, parts of Pennsylvania, Maryland, and New Jersey. The study was approved by ChristianaCare Institutional Review Board.

### 2.2. Data and data preparation

The analysis dataset was extracted from ChristianaCare EHR system and includes only patients diagnosed with COVID-19 admitted to ChristianaCare. To be considered as having the diagnosis of COVID-19, patients must have a positive COVID status informed by the result of COVID-PCR test. We excluded (i) patients who died in the hospital, (ii) patients who had hospital LOS of more than 40 days, and (iii) patients who were not discharged as of August 11, 2020. This cutoff date was selected to provide a 40-day follow-up period for patients admitted on the last day of the study period. For patients with multiple hospitalizations related to COVID-19 within the study period, only the first visit was included.

Demographics assessed were patients’ age, sex, race, and ethnicity. We used the Elixhauser algorithm [20] based on ICD10 codes to identify the patients’ comorbidities. We generated two more variables: Arrival day (weekend vs. weekday), and arrival shift (day 6 AM-6 PM, vs. night).

### 2.3. Statistical analysis

**Figure 1:**
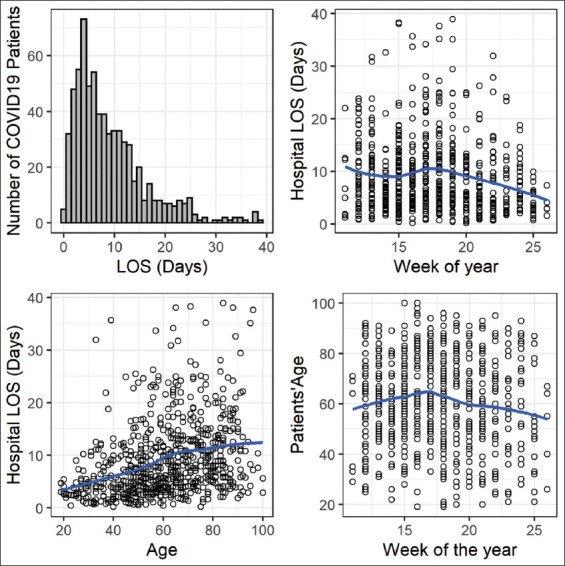
Exploratory graphs: (i) Histogram of hospital LOS (top-left), (ii) scatterplot of the hospital length of stay versus week of the year (top-right), (iii) scatterplot of hospital length of stay versus patients’ age (bottom-left), and (iv) scatterplot of patients’ age versus week of the year (bottom-right)

To describe the patient population, we used counts and percentages for categorical variables, means and standard deviations for normally distributed continuous variables, and medians and interquartile ranges (IQR) for skewed continuous variables. Patients’ disposition from the hospital was defined as an event of interest and followed time-to-event analysis approaches [21]. The patients’ disposition from the hospital was classified as follows: Discharge to home, discharge to hospice, or discharge to skilled nursing or psychiatric facilities. To explore the pattern of LOS overtime, both a histogram (Figure 1) and a locally estimated scatterplot smoothing curve were plotted. Kaplan–Meir curves (probability of patients still being in the hospital at given day after admission) were computed and stratified by sex, age group, race, ethnicity, and comorbidities and differences in Kaplan–Meir curves were tested using log-rank tests.

To evaluate the effect of patients’ demographic and clinical characteristics on the LOS, we utilized a multivariable accelerated failure time (AFT) models with exponential, log-normal, log-logistic, Weibull, and gamma distribution. The best fitting model was selected based on log-likelihood statistics. The AFT model is a survival analysis that directly models the log of time to an event as a function of a vector of model covariates [22] and is fit in three parts. First, we fit an AFT model with patient’s age, sex, race, ethnicity, hospital arrival shift, hospital arrival day, Elixhauser comorbidity count, and week of the year as covariates (Model 1, 8 parameters). Weeks since the onset of pandemic were introduced in the model to account for possible temporal trends in the hospital LOS. Second, we extended Model 1 by introducing one comorbidity at a time (Model 2s), creating a suite of 9-parameter models. The comorbidities incorporated were hypertension, diabetes, obesity, fluid and electrolyte disorder, congestive heart failure, chronic lung disease, coagulopathy, coronary artery disease, renal failure, neurological disorder, and peripheral vascular disease. These comorbidities were extracted through the Elixhauser algorithm and selected based on clinical relevance. Finally, we fit a third model (Model 3) with all the covariates in Model 1 and only comorbidities with *P*<0.25 in Model 2s (Table 1).

**Table 1:** Demographic and clinical characteristics of all the study patients stratified by ICU versus non-ICU utilization

Variable	All patients (n=687)	ICU patients (n=146)	Non-ICU patients (n=541)	*P*-value
Age (Mean, SD)	60.94 (18.10)	60.93 (15.51)	60.94 (18.76)	0.997
Age category				0.031
<50	189 (27.51%)	34 (23.29%)	155 (28.65%)	
50-60	130 (18.92%)	32 (21.92%)	98 (18.11%)	
60-70	134 (19.51%)	37 (25.34%)	97 (17.93%)	
70-80	107 (15.57%)	26 (17.81%)	81 (14.97%)	
More than 80	127 (18.49%)	17 (11.64%)	110 (20.33%)	
Sex				0.025
Female	348 (50.66%)	62 (42.47%)	286 (52.87%)	
Male	339 (49.34%)	84 (57.53%)	255 (47.13%)	
Race				0.882
Black/African-American	307 (44.69%)	64 (43.84%)	243 (44.92%)	
White	267 (38.86%)	56 (38.36%)	211 (39.00%)	
Other	113 (16.44)	26 (17.80)	87 (16.08%)	
Ethnicity				0.776
Hispanic or Latino	103 (14.99%)	21 (14.38%)	82 (15.16%)	
Non-Hispanic or Latino	566 (82.39%)	120 (82.19%)	446 (82.44%)	
Unknown/declined	18 (3.42%)	5 (3.42%)	13 (2.40%)	
Insurance type				0.201
Commercial	246 (35.81%)	59 (40.41%)	187 (34.57%)	
Medicaid	120 (17.47%)	19 (13.01%)	101 (18.67%)	
Medicare	321 (46.72%)	68 (46.58%)	253 (46.77%)	
Arrival day				0.326
Weekday	511 (74.38%)	104 (71.23%)	407 (75.23%)	
Weekend	176 (25.62%)	42 (28.77%)	134 (24.77%)	
Arrival shift				0.173
Day	326 (47.45%)	62 (42.47%)	264 (48.80%)	
Night	361 (52.55%)	84 (57.53%)	277 (51.20%)	
Utilization				
ICU	146 (21.25%)	146 (100.00%)	0	
Ventilator	46 (6.70%)	46 (31.51%)	0	
Elixhauser comorbidity counts (median, IQR)	4 (2, 8)	4 (2, 8)	4 (2, 8)	0.971
Disposition				0.873
Discharged to home	481 (70.01%)	103 (70.55%)	378 (69.87%)	
Discharged to hospice	39 (5.68%)	7 (4.79%)	32 (5.91%)	
Discharged to SNF/rehab/psychiatric facility	167 (24.31%)	36 (24.66%)	131 (24.21%)	
Hospital length of stay in days (median, IQR)	7.18 (3.86, 12.15)	12.34 (8.68, 20.10)	5.72 (3.40, 10.61)	<0.001
ICU length of stay in hours (median, IQR)		3.37 (1.64, 5.47)		

The values are count and percentage unless otherwise noted

COVID-19 patients admitted to the ICU may differ in terms of severity of illness and treatment management. For that reason, similar models of LOS were fit restricted to non-ICU patients. Because of our relatively small sample size, we could not fit separate models for ICU patients. For ease of interpretation, results from the AFT models are presented in terms of time ratio (TR), a ratio of expected LOS between the patients with and without the factor, obtained by exponentiating the model coefficients.

All statistical analyses were done using SAS 9.4® and visualization was done in R. *P*<0.05 was used to determine statistical significance in the analyses.

## 3. Results

### 3.1. Patient characteristics

Among the 815 COVID-19 patients admitted to ChristianaCare before June 30, 2020, 98 (12%) died in the hospital, 26 had LOS longer than 40 days, and four were not discharged as of August 11, 2020. This resulted in a population of 687 patients. Among them, 146 (21%) were admitted to ICU (Table 2). Distributions of patients LOS are provided in Figure 1.

**Table 2:** Results from the accelerated failure time models of the hospital length of stay of ChristianaCare COVID-19 patients

Parameter	Model 1			Model 2s			Model 3		
		
	TR	95% CI of TR	*P*-value	TR	95% CI of TR	*P-*value	TR	95% CI of TR	*P-*value
Age	1.02	(1.01, 1.02)	<0.001				1.02	(1.01, 1.02)	<0.001
Weeks of the year	0.97	(0.96, 0.99)	<0.001				0.97	(0.96, 0.99)	0.000
Male	1.16	(1.04, 1.29)	0.010				1.145	(1.03, 1.28)	0.015
Female (reference)									
Non-Hispanic or Latino	0.92	(0.73, 1.15)	0.454				0.89	(0.71, 1.11)	0.308
Unknown ethnicity	0.66	(0.45, 0.97)	0.035				0.66	(0.45, 0.95)	0.028
Hispanic or Latino (reference)									
Black	0.98	(0.87, 1.12)	0.796				0.99	(0.88, 1.13)	0.927
Other/unknown race	0.99	(0.79, 1.23)	0.901				0.97	(0.78, 1.2)	0.758
White (reference)									
Arrival day (weekend vs. weekday)	1.05	(0.93, 1.19)	0.440				1.05	(0.93, 1.19)	0.432
Arrival shift (night vs. day)	0.98	(0.87, 1.09)	0.667				0.99	(0.88, 1.1)	0.782
Number of comorbidities	1.02	(1.01, 1.03)	0.026				0.99	(0.97, 1.02)	0.564
Hypertension				1.12	(0.77, 1.04)	0.1462	0.91	(0.78, 1.06)	0.212
Fluid and electrolyte disorder				1.20	(1.04, 1.39)	0.0131	1.24	(1.07, 1.43)	0.005
Congestive heart failure				1.12	(0.93, 1.34)	0.2332	1.15	(0.96, 1.38)	0.130
Coagulopathy				1.16	(0.98, 1.38)	0.0931	1.15	(0.97, 1.37)	0.116
Obesity				1.07	(0.94, 1.22)	0.3193			
Diabetes				1.04	(0.91, 1.19)	0.5462			
Chronic lung disease				0.95	(0.83, 1.09)	0.466			
Coronary artery disease				0.91	(0.77, 1.08)	0.2794			
Renal failure				1.06	(0.9, 1.26)	0.4766			
Neurological disorder				1.03	(0.87, 1.2)	0.7587			
Peripheral vascular disease				0.99	(0.83, 1.17)	0.8649			

Model 1 includes age, sex, race, ethnicity, comorbidity counts, and week since onset of pandemic as covariates. Model 2 is separate models with covariates of Model 1 and the given comorbidity as covariates. Model 3: Model1 + Comorbidities with P<0.25 in Model 2s. TR: Time ratio

### 3.2. Results from univariate and bivariate analysis

The distribution of LOS was highly right skewed (Figure 1(i)) with median of 7.18 days (IQR: 3.86-12.15). The median LOS was 12.34 days (IQR: 8.68-20.10) and 5.72 days (IQR: 3.40-10.61) for ICU and non-ICU patients, respectively.

The Kaplan–Meir curve stratified by patients’ age group, sex, race, ethnicity, primary insurance (in cases of dual eligibility), hospital arrival day, and comorbidities is plotted in Appendix 1. The Kaplan–Meir curves plotting the probability of still being in the hospital were significantly different for the strata of age group, race, ethnicity, and insurance. Older patients, White, Non-Hispanic or Latino, and patients covered by Medicare have longer LOS (Appendix 1). Similarly, the Kaplan–Meir curves were also significantly different for patients with and without comorbidities such as hypertension, electrolyte disorder, and diabetes. Patients with those comorbidities have a prognosis of significantly longer LOS (Appendix 1). In the bivariate analysis using Kaplan–Meir curve, the hospital LOS did not differ significantly by patients’ sex, hospital arrival day (weekday vs. weekend), obesity, and chronic lung disease.

The majority (70%) of patients were discharged to home, 24% to skilled nursing or psychiatric facilities, and 6% to hospice. Hospital LOS differed significantly by patients’ disposition. Patients discharged to hospice had the longest LOS with median of 12.1 days (IQR: 7.4-20.8) followed by patients discharged to skilled nursing or psychiatric facilities (Median: 10.7, IQR: 5.2-15.1) and patients discharged to home (median: 5.9, IQR: 3.5-10.5).

### 3.3. Results from multivariable AFT models

The AFT models with a gamma distribution were found to be the best fitting models for LOS (Table 1 and Appendix 2). Patients’ age, sex, number of comorbidities, and weeks since onset of pandemic were significantly associated with LOS. The expected LOS increased by 2% (TR=1.02, CI: 1.01-1.02) with a year increase in age. Similarly, the expected LOS of male patients was 16% longer than that of female patients (TR=1.16, CI: 1.04-1.29). For every additional comorbidity, LOS increased by 2% (TR=1.02, CI: 1.01-1.03). Each consecutive week after the onset of the pandemic is associated with a 3% decrease of LOS (TR=0.97, CI: 0.96-0.99). Patients’ race, ethnicity, hospital arrival day, and hospital arrival shift were not associated with LOS. After adjusting for patents’, sex, race, ethnicity, week of the year, hospital arrival day, arrival shift, and number of comorbidities, the only comorbidity that was significantly associated with LOS was fluid and electrolyte disorder (TR=1.20, CI: 1.04-1.39). The effect estimates for age, sex, race, ethnicity, week of the year, hospital arrival day and shift, and number of comorbidities remained consistent while adjusting for comorbidities (Model 3).

## 4. Discussion

While time of study was concluded in other studies as a covariate that does not impact LOS, we found that each week after the onset of pandemic is associated with decreased LOS. This could be explained by the fact that caregivers have become more experienced and proficient at managing the disease. There were also newer technologies with quicker turn-around time for tests, resulting in a shorter pre-discharge holding time in the hospital. Analysis also shows that the age of hospitalized COVID-19 patients decreases since the pandemic started. Hence, the decrease of LOS is also consistent with the age association with LOS discussed above. It is also likely that the hospital admitted a higher volume of COVID-19 patients who were homeless at the beginning of the pandemic. Due to the unknown impact of discharging homeless patients into the community, care teams took a more conservative approach in ensuring these patients have the appropriate resources to recover in the community. As homelessness data were not well collected, quantification of homeless patients LOS continues to be a challenge. However, patients discharged to hospice and skilled nursing facilities also had higher LOS, likely due to some facilities not prepared to readmit COVID patients due to the lack of appropriate protocol to ensure safety, hence delaying discharges of these patients from the hospital.

The most prevalent comorbidity in our population was hypertension. Contrary to Richardson *et al.*, our next prevalent comorbidities were fluid and electrolytes disorders, and obesity [23]. While fluid and electrolytes disorders could be associated with other variables such as renal failure and age, unadjusted analysis indicated its high prevalence compared to other populations in the literature that were similarly studied. Wang *et al*. [24] found that sex had no effect on LOS. However, we found that there was a statistically significant difference (*P*=0.015) after adjusting for patient clinical and demographic characteristics in the overall COVID-19 population. This could be due to interactions among variables which could be studied in future work. Similarly, adjusted analysis showed every 1-year increase in age is associated with an increase of 2% in LOS (*P*<0.001), as compared to Rees *et al*. (2020). We also performed similar analysis in the subgroup of non-ICU COVID patients. Age, race, ethnicity, comorbidity counts, hospital arrival day, and shift showed a similar effect on LOS. However, sex did not have significant effect on LOS of non-ICU patients (Appendix 2).

Rees *et al*. [13] also concluded that disease severity was not associated with LOS while our analysis showed, using the definition of count of comorbidities as a proxy for disease severity, every one increase in comorbidity count is associated with an increase in LOS by 2% (*P*<0.05) in the overall and non-ICU populations, in Model 1.

As mentioned, our study shows an association of older age with longer LOS. In the study site, geriatric patients diagnosed with COVID-19 are cohorted with other COVID-19 patients. As geriatric patients’ medical needs could differ from other patients, such practice could make best practice for geriatric care more challenging.

In our study period, the first case of COVID-19 was at the end of the flu season. Hence, data were not available for study team to investigate seasonality impact on COVID-19 patients LOS, especially those who could be infected with the flu and COVID-19 simultaneously. We also do not explore the implications of COVID-19 patients LOS to hospital costs based on various patient types as costs are not in scope to this study.

Methodologically, methods such as random forest and bootstrapping could be used to further enhance and complement the methods applied in this study.

Although LOS is an important factor in predicting hospital resource needs in many models, it is rarely a primary outcome from studies. While there are some similarities, our study shows that the LOS of our patients, as well as the factors associated with it, varies from that published in the literature. This discrepancy is not surprising due to the still emerging understanding of the disease. However, it does illustrate the need for institutions that either utilize universal COVID-19 predictive models or custom-developed models to conduct further studies to understand their local populations to use the appropriate LOS values in their census prediction, as LOS is a key driver to the predicted values.

### 4.1. Limitations

As this study is a single site study with limited sample size, the analysis only shows patients admitted to ChristianaCare and may not be representative of COVID-19 patient characteristics and LOS for other hospitals. The dataset with relatively modest sample size was collected retrospectively and could have included confounding factors that introduced biases to the analysis. That said, investigation of hospital LOS will assist in enhancements of predictions models for local jurisdictions and need not be generalizable.

A portion of literature supporting this work is based on studies published in MedRxiv which has not been peer-reviewed. However, we think that it is relevant to utilize carefully selected literature from MedRxiv as this is an area that is rapidly expanding beyond the traditional publication time frames of many peer-reviewed journals. As a result, we also utilized articles from peer-reviewed journals to complement MedRxiv to reduce bias that might be present.

## Figures and Tables

**Appendix 1:**
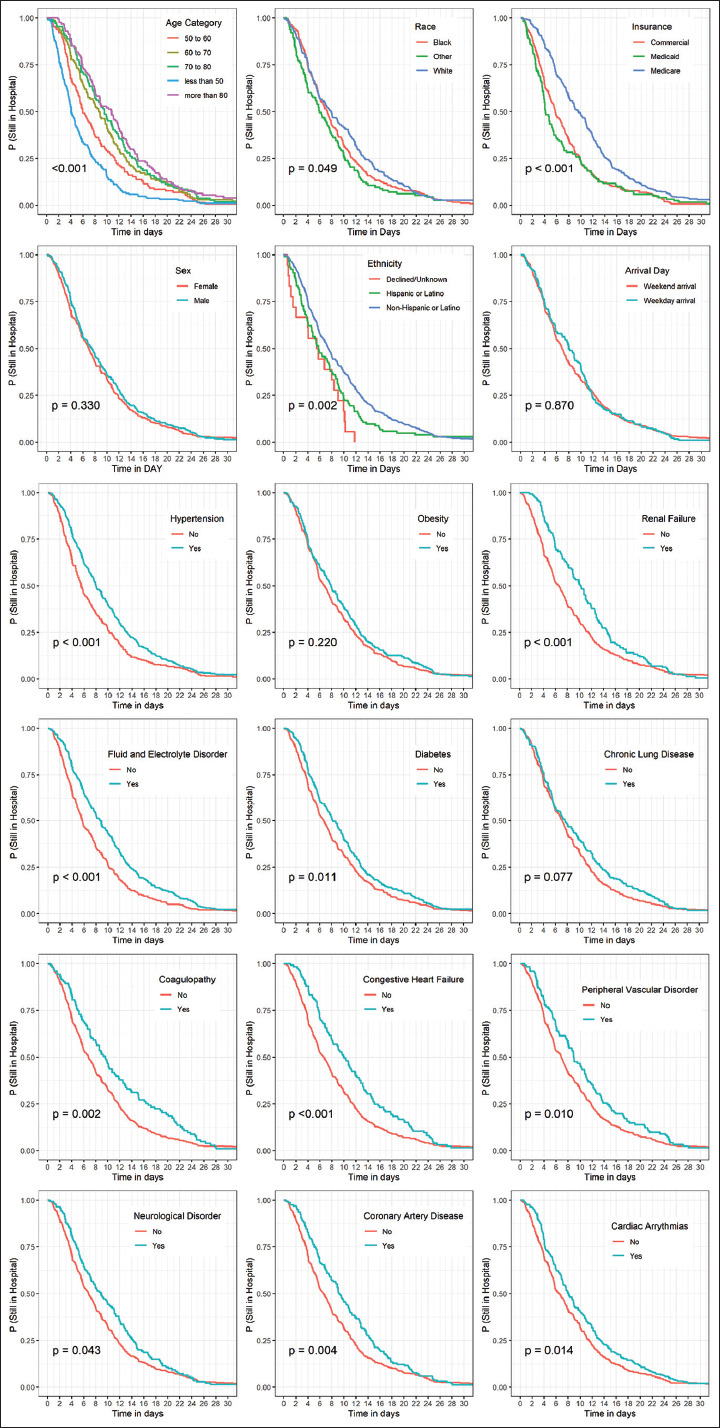
Kaplan–Meir curve of probability of still being in the hospital at a given day after admission stratified by demographics and clinical characteristics

## Appendix

**Appendix 2:** Results from the accelerated failure time models of the hospital length of stay of the non-ICU COVID-19 patient

Parameter	Model 1			Model 2s			Model 3		
		
	TR	95% CI of TR	*P*-value	TR	95% CI of TR	*P*-value	TR	95% CI of TR	*P*-value
Age	1.02	(1.01, 1.02)	<0.001				1.02	(1.01, 1.02)	<0.001
Weeks of the year	0.99	(0.96, 0.99)	<0.004				0.98	(0.96, 0.99)	0.004
Male	1.08	(0.96, 1.21)	0.215				1.05	(0.94, 1.19)	0.376

TR: Time ratio

## References

[1] Zhu N, Zhang D, Wang W, Li X, Yang B, Song J (2020). A Novel Coronavirus from Patients with Pneumonia In China, 2019. N Engl J Med.

[2] Mallapaty S (2020). Scientists Fear Coronavirus Spread in Vulnerable Nations. Nature.

[3] Gorbalenya AE, Baker SC, Baric RS, de Groot RJ, Drosten C, Gulyaeva AA (2020). The Species Severe Acute Respiratory Syndrome-related Coronavirus:Classifying 2019-nCoV and Naming it SARS-CoV-2. Nat Microbiol.

[4] Omer SB, Malani P, del Rio MC (2020). The COVID-19 Pandemic in the US A Clinical Update. JAMA.

[5] WHO Director-General's Opening Remarks at the Media Briefing on COVID-19 11 March 2020.

[6] Johns Hopkins University and Medicine Coronavirus Resource Center:COVID-19 Case Tracker.

[7] Centers for Disease Control and Prevention New Variants of the Virus that Causes COVID-19.

[8] Abir M, Nelson C, Chan EW, Al-Ibrahim H, Cutter C, Patel K (2020). RAND Critical Care Surge Response Tool:An Excel-Based Model for Helping Hospitals Respond to the COVID-19 Crisis. RAND Crit Care Surge Response Tool An Excel Model Help Hosp Respond to COVID-19 Cris.

[9] Qventus Localized COVID-19 Model and Scenario Planner.

[10] Alvarez MM, Gonzalez-Gonzalez E, Santiago GT (2020). Modeling COVID-19 Epidemics in an Excel Spreadsheet:Democratizing the Access to First-hand Accurate Predictions of Epidemic Outbreaks. medRxiv.

[11] Becker M, Chivers C (2020). Announcing CHIME. A Tool for COVID-19 Capacity Planning. Predict Heathcare.

[12] Rodrigues HS (2016). Application of SIR Epidemiological Model:New Trends.

[13] Rees EM, Nightingale ES, Jafari Y, Waterlow NR, Waterlow S, Pearson CA (2020). COVID-19 Length of Hospital Stay:A Systematic Review and Data Synthesis. medRxiv.

[14] Librero J, Peiró S, Ordiñana R (1999). Chronic Comorbidity and Outcomes of Hospital Care:Length of Stay, Mortality, and Readmission at 30 and 365 Days. J Clin Epidemiol.

[15] Kaplan V, Angus DC, Griffin MF, Clermont G, Watson RS, Linde-Zwirble WT (2002). Hospitalized community-acquired pneumonia in the elderly:Age-and sex-related patterns of care and outcome in the United States. Am J Respir Crit Care Med.

[16] https://vizhub.healthdata.org/gbd-compare/.

[17] Dynamics UND of E and SAP (2019). World Population Prospects.

[18] Fine MJ, Pratt HM, Obrosky DS, Lave JR, McIntosh LJ, Singer DE (2000). Relation between Length of Hospital Stay and Costs of Care for Patients with Community-acquired Pneumonia. Am J Med.

[19] White BA, Biddinger PD, Chang Y, Grabowski B, Carignan S, Brown DF (2013). Boarding Inpatients in the Emergency Department Increases Discharged Patient Length of Stay. J Emerg Med.

[20] Quan H, Sundararajan V, Halfon P, Fong A, Burnand B, Luthi JC (2005). Coding Algorithms for Defining Comorbidities in ICD-9-CM and ICD-10 Administrative Data. Med Care.

[21] Hougaard P (1999). Fundamentals of Survival Data. Biometrics.

[22] Collett D (2015). Modelling Survival Data in Medical Research.

[23] Richardson S, Hirsh JS, Narashimhan M (2020). Presenting Characteristics, Comorbidities, and Outcomes among 5700 Patients Hospitalized with COVID-19 in the New York City Area. JAMA.

[24] Wang Z, Ji JS, Liu Y, Liu R, Zha Y, Chang X (2020). Survival Analysis of Hospital Length of Stay of Novel Coronavirus (COVID-19) Pneumonia Patients in Sichuan, China. medRxiv.

